# A new Mach–Zehnder interference temperature measuring sensor based on silica-based chip

**DOI:** 10.1038/s41598-024-59447-z

**Published:** 2024-04-15

**Authors:** Guoqiang Li, Tao Li, Yongfang Liu, Yuanjin Zheng

**Affiliations:** 1 School of Electrical and Electronic Engineering, Nanyang Technological University, Nanyang Avenue, Singapore, 639798 Singapore; 2 Institute of Novel Semiconductors, Institute of Crystal Materials, State Key Laboratory of Crystal Materials, Shandong University, Jinan, 250100 China; 3 Shanghai Advanced Research Institute, Chinese Academy of Sciences, Shanghai, 201204 China

**Keywords:** Optical sensors, Silicon photonics

## Abstract

A new type of silicon-based Mach–Zehnder interference (MZI) temperature sensor chip with “mosquito coil” structure was designed. The sensor chip used a new MZI interference structure. After the light entered the chip, it split and interfered in the combiner of the chip. The change in the surrounding temperature will cause the refractive index of the waveguide to change, which will cause the output light intensity to change. The sensor used a frequency stabilized laser that was based on a Bragg grating fiber. The experimental results showed that this structure could achieve a resolution of 0.002 °C and measuring range of 30 °C.

## Introduction

The production and research activities in the marine field have a large demand for sensors, especially marine pastures^1–4^, such as temperature sensor^5,6^, salinity sensor^7^, pH sensor^8^, dissolved oxygen sensor, turbidity sensor, special organic sensor and so on^9^. It is used to obtain the environmental elements and water quality information of the marine pasture. It is of great significance to ensure the ecological stability of the marine pasture, prevent pollution of water bodies and maintain efficient production activities. In addition to some intertidal organisms that are often exposed to air and submerged in seawater, other marine organisms tend to live in relatively stable temperatures and are sensitive to temperature changes^10^. At the same time, temperature changes not only affect the survival, reproduction and development of organisms, but also interact with other environmental factors, such as dissolved oxygen^11^, salinity and other environmental factors. Therefore, monitoring temperature changes is of great significance for maintaining the ecological stability of marine pastures and maintaining efficient production activities. At present, the existing temperature measurement methods mainly include platinum resistance temperature sensor^12–14^, fiber temperature sensor^15,16^, optical waveguide temperature sensor^17,18^, thermocouple temperature sensor^19^ and so on. The traditional platinum resistance temperature sensor has stable chemical properties, good linearity, good stability and high pressure resistance, but has the disadvantages of slow thermal response, poor resistance to vibration and impact, and high price. It is not suitable for marine pastures temperature measurement. Optical temperature sensors are mainly fiber temperature sensors and optical waveguide temperature sensors. The fiber temperature sensor mainly has three types of temperature sensors based on fiber FP cavity^20,21^, fiber grating^22,23^, and photonic crystal fiber^24–26^. Among them, the length of the fiber FP cavity changes with temperature, which causes the peak of the output spectrum to drift with temperature. It is small in size, high in precision, and fast in heat, but the FP cavity is difficult to manufacture. The fiber Bragg grating temperature sensor has small volume, light weight, low cost and mature technology, but its impact resistance and vibration resistance are poor, and the influence of microorganisms cannot be avoided, which will affect the measurement results.

The research on marine pastures includes research on environmental control technology. It monitors environmental factors and water quality information through various types of sensors, sends information to the upper computer through cable analysis and judgment, and regulates the marine pasture through negative feedback mechanism to ensure the production activities are efficient and stable. With the development of marine technology, marine pastures put forward higher requirements for in-situ real-time measurement of various physical quantities.

As far as we know, the existing optical waveguide temperature sensors based on MZI interference structure have the highest accuracy^27–32^, but their sensitive areas are small, the sensitivity to external temperature changes is low, and the response time is long. Existing optical waveguide temperature sensors mainly use polymer materials^33–35^ such as SU-8, PDMS, PMMA and the like. However, polymer materials are easily decomposed in seawater environments^36,37^, making it difficult to meet the needs of in situ measurements in marine pastures. Therefore, the demand for low-cost, high-precision temperature sensors has become more and more urgent. In recent years, silicon-based optoelectronic technology has matured, and all-optical sensing components and their hybrid integration technologies have become more and more mature. The optical waveguide temperature sensor has small volume, low cost, immune electromagnetic interference, and can be used in various extreme environments, and has become a research hotspot of high-precision temperature sensors in recent years.

In this paper, we proposed a waveguide temperature sensor based on MZI, which adopted a “mosquito coil” structure to grow sensitive area and increase the sensitivity. Finally, an accuracy of 0.01 °C and a temperature resolution of 0.002 °C were achieved. Compared with traditional sensors, our proposed spiral structure is smaller in size, lower in cost, easier to install, and can be integrated with detectors, optical switches and other devices. At the same time, we designed a fiber Bragg grating frequency-stabilized laser, which provides a light source with a stable frequency and light intensity for the waveguide.

## Design and analysis

### Mach–Zehnder interference with mosquito structure

The measurement principle is Mach–Zehnder interference. For the optical waveguide MZI, the optical path difference between the sensing arm and the control arm is the main parameter that affects the sensitivity of the sensor, and the optical path difference is related to the refractive index and length. We used SiO_2_ as the core layer of the optical waveguide with a thermo-optic effect coefficient $dn/dT$ of -0.192 × 10^–6^/°C and a thermal expansion coefficient $dl/dT$ of 0.45 × 10^–6^/°C^38^. Without considering the loss, the output light intensity of MZI is:

1 $${I}_{out}=\frac{1}{2}{I}_{0}(1+cos\Delta \varphi )$$

The phase difference between the two arms of the MZI is:

2 $$\Delta \mathrm{\varphi }=\frac{2\pi }{\lambda }{n}_{eff}({L}_{1}-{L}_{2})$$

where ∆φ is the phase difference, λ is the laser wavelength, ${n}_{eff}$ is the effective refractive index of the waveguide core, ${L}_{1}$ is the length of the sensing arm, and ${L}_{2}$ is the length of the reference arm. It can be seen that the factors that affect the phase difference include the laser wavelength, the effective refractive index of the core, and the length difference between the arms.

When the laser wavelength is stable, the phase difference change due to temperature is:

3 $$\frac{d\Delta \varphi }{dT}=\frac{2\pi }{\lambda }\left\{\frac{d{n}_{eff}}{dT}\left({L}_{1}-{L}_{2}\right)+{n}_{eff}\frac{d({L}_{1}-{L}_{2})}{dT}\right\}$$

**Figure 1:**
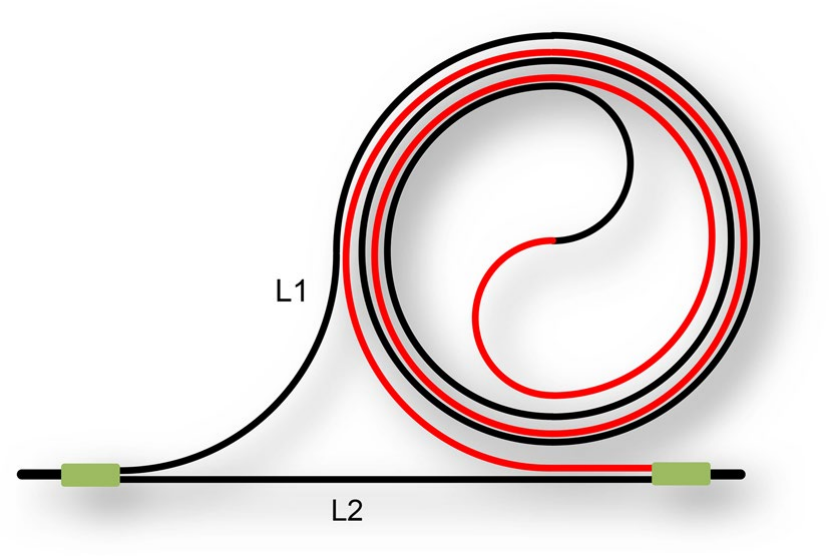
MZI mosquito coil structure. The light beam enters from the left and enters L1 and L2 respectively after splitting. The beam propagated counterclockwise in the red part of L1 and passes clockwise in the black part. Finally, it was combined at the beam combiner.

Therefore, when the temperature changes, it will result in a change in the emitted light intensity, and the temperature can be demodulated by the change in light intensity. According to the above formula, one of the ways to increase the sensitivity of MZI is to increase the length of the sensing arm. In this paper, a mosquito-repellent-type structure of MZI is designed. Figure 1 shows the MZI structure we designed.

At the incident end of the MZI structure, the light splits into two beams, one beam passing through the sensing arm and the other beam passing through the reference arm as reference light. At the end of the MZI, the two beams combine to interfere. Finally, the interference is detected by the light intensity detector. The sensor arm in the MZI structure is a mosquito coil structure that minimizes transmission loss while maximizing the length of the sensing arm. The measuring range we designed was from -5 °C to -35 °C (the range of seawater temperature variation). Within this range, the two beams in the sensor combiner have a phase difference of π, or a wavefront difference of two beams of λ/2. That is the following formula:

4 $$\frac{{L}_{1}-{L}_{2}}{{\lambda }_{{T}_{2}}}\cdot \left({\lambda }_{{T}_{1}}-{\lambda }_{{T}_{2}}\right)=\frac{{\lambda }_{{T}_{1}}}{2}$$

Thus the length of the sensor arm should be:

5 $${L}_{1}=\frac{{\lambda }_{{T}_{2}}{\lambda }_{{T}_{1}}}{2({\lambda }_{{T}_{2}}-{\lambda }_{{T}_{1}})}+{L}_{2}$$

where ${L}_{1}$ is the length of the sensing arm, ${L}_{2}$ is the length of the reference arm, ${\lambda }_{{T}_{2}^\circ {\text{C}}}$ is the laser wavelength at ${T}_{2}$ °C, and ${\lambda }_{{T}_{1}^\circ {\text{C}}}$ is the laser wavelength at ${T}_{1}$ °C.

In order to ensure the interference of the beam in the MZI and in order to obtain a high extinction ratio and low propagation loss, only a single mode should be transmitted in the core layer of the planar waveguide. The optoelectronic device herein used SiO_2_ as a substrate on which a waveguide layer was formed. We set the waveguide to a rectangular waveguide with a refractive index difference of 0.0298 between the core and the cladding. In a slab optical waveguide, the mode of actual propagation can be calculated by the following formula:

6 $${k}_{x}{\text{w}}=\mathrm{p\pi }-2{{\text{tan}}}^{-1}{k}_{x}\frac{1}{\sqrt{{\left\{\frac{2\pi \sqrt{{n}_{1}^{2}-{n}_{2}^{2}}}{\lambda }\right\}}^{2}-{k}_{x}}}$$

7 $${k}_{y}h=q\pi -2{tan}^{-1}\frac{{n}_{2}^{2}}{{n}_{1}^{2}}{k}_{y}\frac{1}{\sqrt{{\left\{\frac{2\pi \sqrt{{n}_{1}^{2}-{n}_{2}^{2}}}{\lambda }\right\}}^{2}-{k}_{y}}}$$

8 $${k}_{z}=\sqrt{{k}_{i}^{2}-{k}_{x}^{2}-{k}_{y}^{2}}$$

9 $${k}_{i}=\frac{2\pi }{\lambda }{n}_{i} (\mathrm{where\, }{n}_{i}\, \mathrm{is\, one\, of\, }{n}_{1}, {n}_{2})$$

where $w$ is the length of the waveguide and $h$ is the width of the waveguide,${n}_{1}, {n}_{2}$ are the refractive indices of the core layer and the cladding layer, respectively.${k}_{x}$, ${k}_{y}$, and ${k}_{z}$ are propagation constants in the $x$, $y$ and $z$ axis directions, respectively.

**Figure 2:**
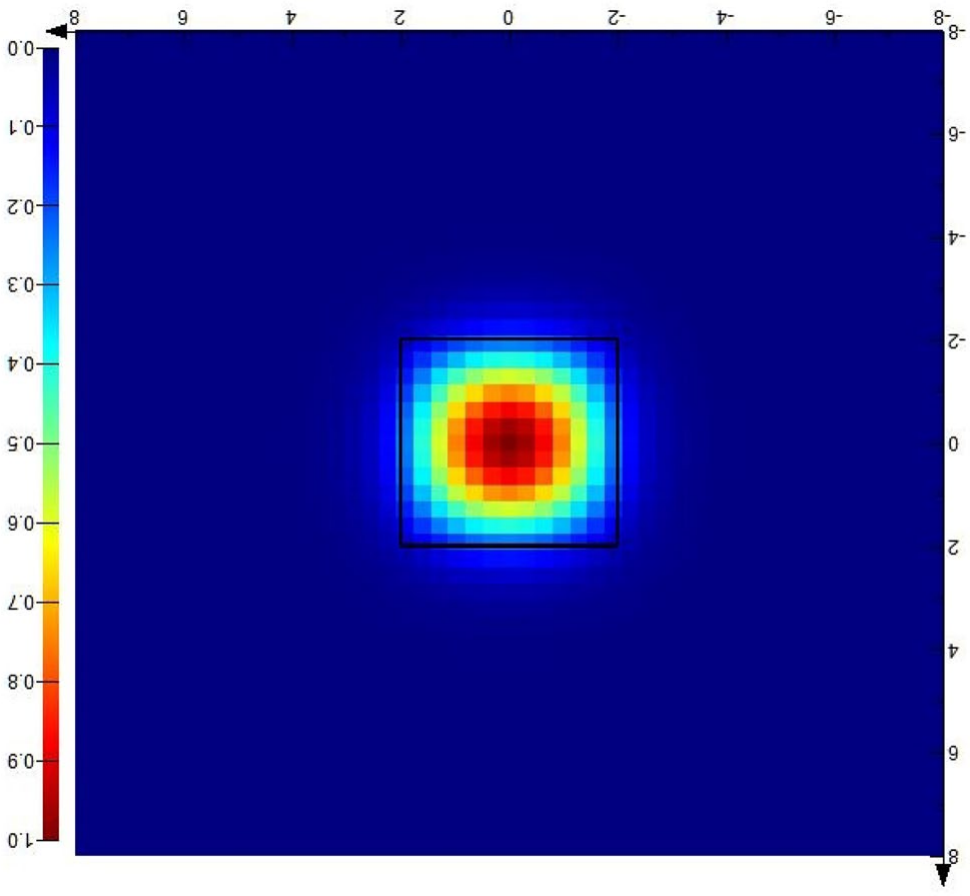
Light propagation mode in the waveguide core.

Through simulation, it was verified that the single-mode light propagated in the core waveguide at this time, and the result is shown in Fig. 2.

The waveguide core layer has a cross-sectional dimension of 3 × 3 μm, wherein the cladding refractive index is 1.4448 and the core refractive index is 1.4746.

### Manufacturing of Mach–Zehnder interference

**Figure 3:**
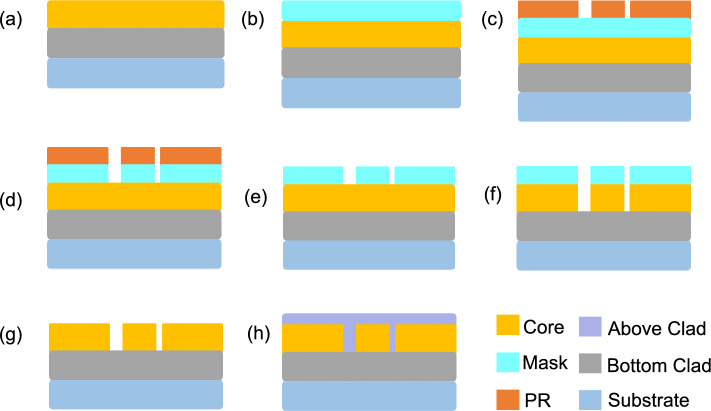
The manufacturing process of Mach Zehnder interference chip.

The Mach–Zehnder interference optical waveguide chip is processed by Shijia Photon Technology Co., Ltd. The solution uses SiO_2_ substrate to form a waveguide layer on it. Figure 3 shows the manufacturing process of optical waveguides.

In Fig. 3, the formation of Core&Bottom Clad in step (a) is a key step in the manufacturing of optical waveguide chips, mainly including material selection, coating, curing, and subsequent processing. Firstly, it is necessary to select suitable optical materials and bottom coating materials. The selection of these materials is based on factors such as their optical properties, thermal stability, mechanical strength, and processing performance. For the Core layer, it is usually necessary to have a high refractive index to effectively guide light waves; The Bottom Clad layer needs to have a lower refractive index to ensure the propagation of light waves in the Core layer. Here, SiO2 based waveguides are used, with substrate refractive index of 1.4444 and cladding of 1.4421. Next, these two materials are respectively coated on the substrate through coating technology. During this process, it is necessary to control the uniformity and thickness of the coating to ensure the performance of the optical waveguide. After coating, it is necessary to perform thermal curing treatment on the material to form a stable structure on the substrate. Finally, perform subsequent processing on the formed Core&Bottom Clad, including cleaning, inspection, and leveling. These processing steps aim to eliminate possible defects and contamination during the processing, and improve the quality and reliability of optical waveguides.

In step (b), make a mask. Mask is a printing like process that plays a role in protecting sensitive areas in chip manufacturing. In step (c), apply photoresist (PR) and ensure uniform coverage of the photoresist through spin coating technique. Ultraviolet light is irradiated onto the photoresist through the mask of the lithography machine, forming an exposure area corresponding to the design pattern. After exposure, the silicon wafer undergoes a development process. The developer will dissolve the photoresist portion that has been exposed to ultraviolet light, while the unexposed portion is retained. In this way, the graphics of the chip design will be displayed on the photoresist. In step (d), perform the etching process. The etching solution will dissolve the unprotected portion of the silicon wafer by the photoresist, forming a depression on the Mask that corresponds to the design pattern. In step (e), clean and inspect to remove photoresist residue and impurities generated by etching. In step (f), the Hard mask is used as the protective layer to etch the Core layer, accurately removing unnecessary Core materials, and forming the desired optical waveguide structure. The advantage of using Hard mask to etch the Core layer is that it can provide higher etching accuracy and stability, especially suitable for the manufacturing of complex and fine optical waveguide structures. In step (g), peel off the hard mask. Before starting the stripping process, ensure that the working environment is clean and dust-free to avoid introducing additional pollution or damage. In step (h), Above clad is formed. Covering and protecting the already formed waveguide structure, ensuring stable transmission of light waves within the waveguide, while preventing the influence of external environment on the performance of the waveguide. The upper cladding material here is SiO2. To ensure the transmission efficiency of light waves within the upper cladding, it is necessary to smooth the surface of the upper cladding. After completing the formation of the upper layer, thoroughly clean the chip to remove any residues and pollutants that may occur during the deposition process. Finally, the manufactured chips are tested.

### Frequency stabilization system

**Figure 4:**
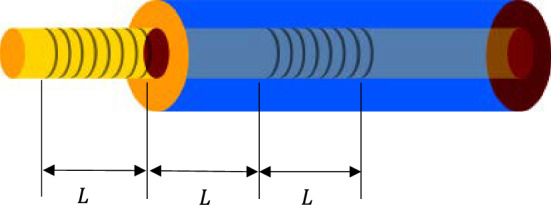
The structure of FBG-FP.

This system used the jitter frequency method to stabilize the laser frequency, and used a FBG-FP to obtain the feedback signal. As shown in Fig. 4, a temperature-sensitive FBG-FP is designed, with two FBGs of the same length, and the length between the two FBGs is the same as the length $L$ of FBG.

FP cavity has a frequency selection function, so it is widely used in laser frequency stabilization systems. In this paper, two FBGs of the optical fiber are used to form an FBG-FP. The optical fiber sensor adopts the FBG-FP structure, in which the FBG length $L$ is 2 cm, the FP length $L$ is 2 cm, and the FP is between the two FBGs. The reflectivity of the FBG is 99.1%. Due to the high reflectivity of the FBG, the FP cavity has a high degree of fineness and a narrow spectrum width. The FBG-FP structure of the optical fiber uses acrylate as the coating layer, and its coefficient of thermal expansion $\mathrm{\alpha }$ is 85 × 10^–6^/°C. According to the transmission matrix theory, the transmission matrix of FBG-FP is^39,40^:

10 $${T}_{FBG-FP}=\langle \begin{array}{cc}{F}_{11}\cdot {F}_{11}\cdot {e}^{-i\eta L}+{F}_{12}\cdot {F}_{21}\cdot {e}^{i\eta L}& {F}_{11}\cdot {F}_{12}\cdot {e}^{-i\eta L}+{F}_{12}\cdot {F}_{22}\cdot {e}^{i\eta L}\\ {F}_{21}\cdot {F}_{11}\cdot {e}^{-i\eta L}+{F}_{22}\cdot {F}_{21}\cdot {e}^{i\eta L}& {F}_{21}\cdot {F}_{12}\cdot {e}^{-i\eta L}+{F}_{22}\cdot {F}_{22}\cdot {e}^{i\eta L}\end{array}\rangle$$

where $\eta =\frac{2\pi {n}_{eff}}{{\lambda }_{b}}$, ${n}_{eff}$ is the effective refractive index of the core and $L$ is the length of FP.

11 $$\left\{\begin{array}{c}{F}_{11}=cosh(\sqrt{{\alpha }^{2}-{\beta }^{2}}L)-i(\frac{\beta }{\sqrt{{\alpha }^{2}-{\beta }^{2}}})\cdot sinh(\sqrt{{\alpha }^{2}-{\beta }^{2}}L)\\ \begin{array}{c}{F}_{12}=i(\frac{\alpha }{\sqrt{{\alpha }^{2}-{\beta }^{2}}})\cdot sinh(\sqrt{{\alpha }^{2}-{\beta }^{2}}L)\\ \begin{array}{c}{F}_{21}=-i(\frac{\alpha }{\sqrt{{\alpha }^{2}-{\beta }^{2}}})\cdot sinh(\sqrt{{\alpha }^{2}-{\beta }^{2}}L)\\ {F}_{22}=cosh\left(\sqrt{{\alpha }^{2}-{\beta }^{2}}L\right)+i(\frac{\beta }{\sqrt{{\alpha }^{2}-{\beta }^{2}}})\cdot sinh(\sqrt{{\alpha }^{2}-{\beta }^{2}}L)\end{array}\end{array}\end{array}\right.$$

12 $$\alpha =\frac{\pi \vartheta }{\lambda }\overline{\Delta {n}_{eff}}\left(z\right)$$

13 $$\beta =\alpha +\delta -d\varphi (z)/2dz$$

14 $$\delta =2\pi {n}_{eff}(\frac{1}{\lambda }-\frac{1}{{\lambda }_{b}})$$

where $\overline{\Delta {n}_{eff}}\left(z\right)$ is the modulation depth of the refractive index, $\Lambda$ is the grid period of the fiber grating,${n}_{eff}$ is the effective refractive index, and $\varphi \left(z\right)$ is the grating chirp, $\vartheta$ is the visibility of fringe modulated by refractive index, $\alpha$ and $\beta$ are the coupling coefficients which are constants, $L$ is the length of FBG, ${\lambda }_{b}$ is the center wavelength of FBG.

According to the above formula, the reflection coefficient and transmission coefficient of FBG-FP can be obtained as:

15 $${r}_{FBG-FP}=\frac{1}{{F}_{11}\cdot {F}_{11}\cdot {e}^{-i\eta L}+{F}_{12}\cdot {F}_{21}\cdot {e}^{i\eta L}}$$

16 $${t}_{FBG-FP}=\frac{{F}_{21}\cdot {F}_{11}\cdot {e}^{-\eta L}+{F}_{22}\cdot {F}_{21}\cdot {e}^{i\eta L}}{{F}_{11}\cdot {F}_{11}\cdot {e}^{-i\eta L}+{F}_{12}\cdot {F}_{21}\cdot {e}^{i\eta L}}$$

Therefore, the reflectance and transmittance of FBG-FP are:

17 $${R}_{FBG-FP}={\left|{r}_{FBG-FP}\right|}^{2}$$

18 $${T}_{FBG-FP}={\left|{t}_{FBG-FP}\right|}^{2}$$

Because the fiber Bragg grating only reflects the incident light wave within the grating bandwidth, the high reflectivity mirror can reflect the light wave in the entire incident wavelength range. Therefore, the FBG-FP cavity is better than the ordinary FP cavity in terms of frequency selection.

19 $${T}_{FBG-FP}={\left|{t}_{FBG-FP}\right|}^{2}=\frac{1}{1+R{sin}^{2}(\frac{2\pi {n}_{eff}L}{\lambda }-{\phi }_{r})}$$

Among them, $R$ is the reflection coefficient of FBG-FP, and ${\phi }_{r}$ is the phase factor of FBG-FP.

**Figure 5:**
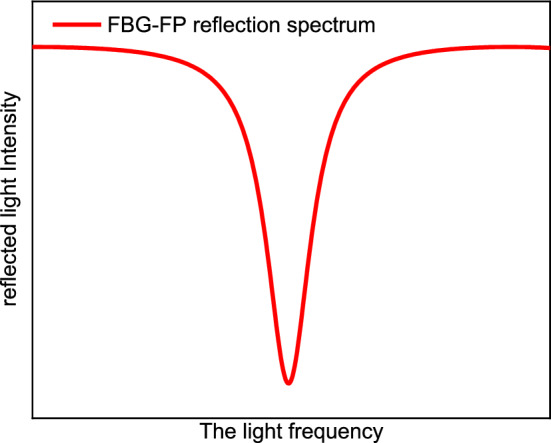
The input frequency changes cause the reflected light intensity to change.

The light intensity reflection spectrum of FBG-FP can be calculated by the above formula. The reflected light intensity of FBG-FP and the input light frequency have the relationship shown in Fig. 5.

**Figure 6:**
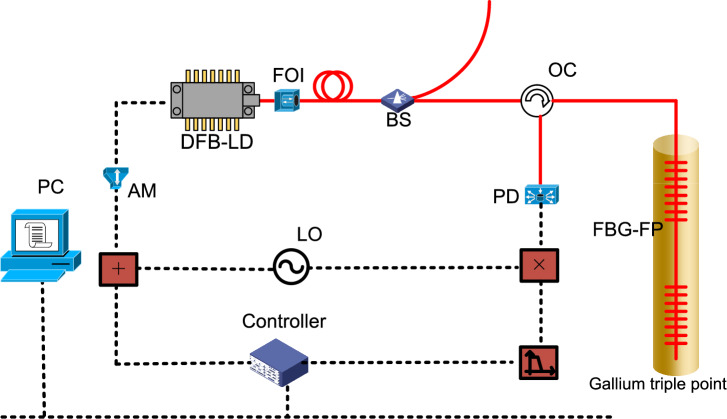
Frequency stabilization system, *DFB-LD* distributed-feedback laser, *FOI* fiber optics isolator, *BS* beam splitter, *OC* optical circulator, *PD* optical detector, *FBG* fiber Bragg grating, *LO* local oscillator, *AM* amplifier.

In the reflected light intensity spectrum of FBG-FP, each trough corresponds to a unique frequency. Therefore, as long as the reflected light intensity is kept at the trough, the output frequency of the laser can be stabilized. Using this feature, we can use the jitter frequency method to stabilize the output frequency of the laser on the reference cavity. The frequency stabilization system is shown in Fig. 6.

The frequency stabilization system includes laser source, fiber sensor and controller. The DFB-LD uses 1550 nm wavelength, and the fiber sensor is used to provide error signal. The fiber optic sensor is placed in a triple point bottle of gallium to obtain a stable environment. So that the various parameters of FBG-FP can maintain a constant value, that is to say, the optical frequency corresponding to the reflection spectrum trough of FBG-FP is fixed.

The laser emitted by the DFB-LD passed through the isolator and was divided into two beams by the fiber beam splitter, one of which was used as the output light, and the other was input into the FBG-FP through the circulator to stabilize the output light frequency of the laser. The reflected light of the FBG-FP was detected by the photodetector after passing through the circulator, and the detection signal was multiplied by the local oscillator (LO). After passing through the low-pass filter, it was transmitted to the controller as an error signal, and the controller calculated the control signal by the PID control algorithm. The control signal and the local oscillator (LO) were added together and transmitted to the DFB-LD to stabilize the frequency of the DFB-LD. The controller and other parameters could be controlled by PC.

Figure 6 shows the structure and working process of PID control.

**Figure 7:**
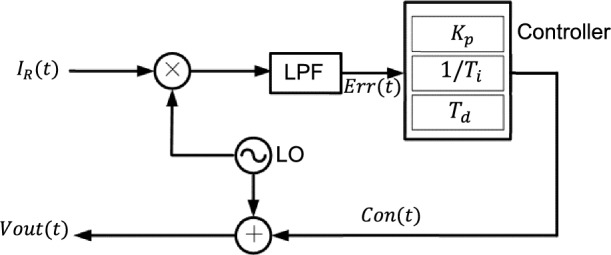
PID controller structure.

As shown in Fig. 7, the reflected signal ${I}_{R}(t)$ of the FBG-FP was multiplied by the local oscillator signal (LO) and then passed through the low-pass filter to obtain the error signal $Err(t)$.

The laser is controlled by the output signal $Vout\left(t\right)$ of the controller:

20 $$Vout\left(t\right)=Con\left(t\right)+LO, LO={\text{sin}}(\omega t)$$

21 $${I}_{R}\left(t\right)={I}_{0}(Vout(t))\cdot {T}_{FBG-FP}({f}_{Vout\left(t\right)}(t))$$

22 $${I}_{R}\left(t\right)\cdot LO={I}_{0}(Vout(t))\cdot {T}_{FBG-FP}({f}_{Vout\left(t\right)}(t))\cdot {\text{sin}}(\omega t)$$

$Err(t)$ is the signal passed through the low-pass filter, which filters out fundamental wave and its harmonic signals, leaving only the DC control signal.

The relationship between the control signal output by PID control and the error signal $Err\left(t\right)$ is:

23 $$Con(t)=Kp(Err\left(t\right)+\frac{1}{Ti}\int Err\left(t\right)dt+Td\frac{dErr(t)}{dt})$$

where ${K}_{p}$ is the proportional coefficient, ${K}_{p}/{T}_{i}$ is the integral coefficient, and $Kp\cdot Td$ is the differential coefficient.

## Results

**Figure 8:**
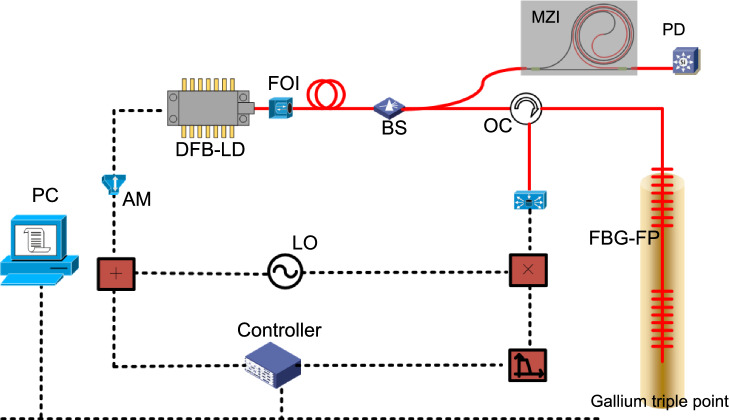
Schematic diagram of slab optical waveguide experiment, *MZI* MZI optical chip.

As shown in Fig. 8, the frequency stability laser was connected to the waveguide temperature sensor via an optical fiber. The output fiber of the optical waveguide was connected to the light intensity detector to record the change of the light intensity with temperature. The optical waveguide temperature sensor was placed in a water bath to measure the temperature of the water.

**Figure 9:**
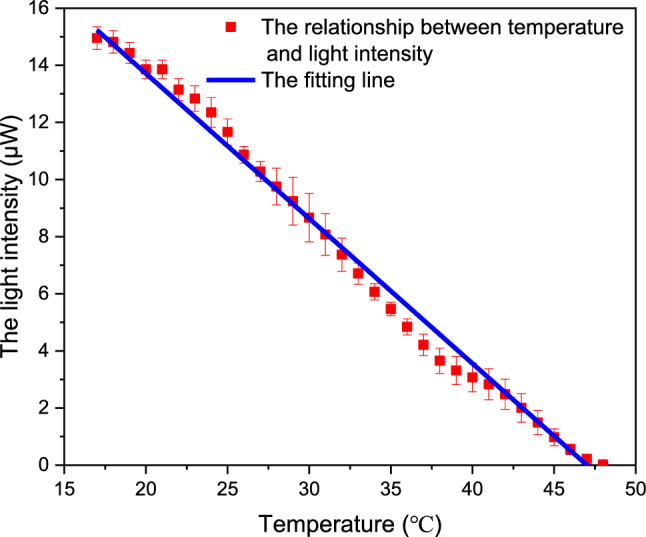
Light intensity changes with temperature during natural cooling.

Figure 9 shows the results measured by the optical waveguide temperature sensor. First, the water was heated to 47 °C by a constant temperature water bath and then cooled to 17 °C. The output light intensity of the MZI was recorded by a light intensity detector.

As can be seen from Fig. 9, as the temperature decreases unilaterally, the light intensity increases unilaterally. When the temperature changed from 47 to 17 °C, the light intensity changed from 0 to 15 μW, which was consistent with theoretical analysis. The resolution of photoelectric detection is 0.1 nw and the accuracy is 1 nW. During the experiment, the temperature changed by 30 °C, resulting in the light intensity changed by 15 μW, so the accuracy of our MZI is 0.002 °C.

**Figure 10:**
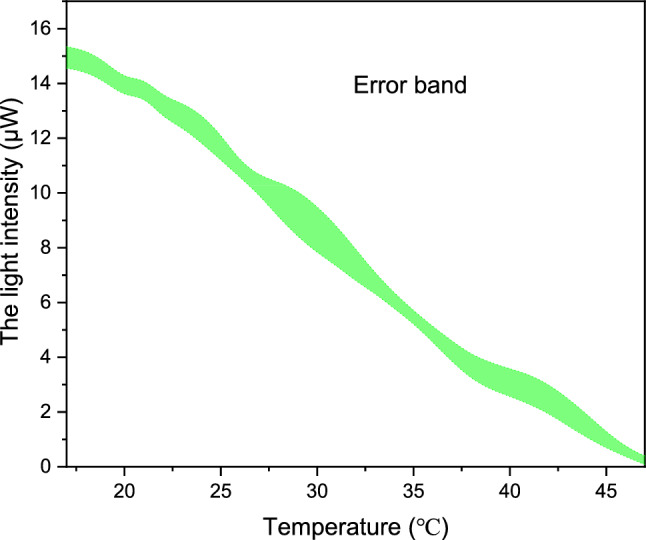
MZI temperature sensor compared with thermometer.

As can be seen from Fig. 10, error bars in 10 repeated measurements. In 10 repeated tests, the error of MZI is larger, which is mainly affected by the instability of the test environment.

## Discussion and conclusion

In conclusion, we designed an optical waveguide temperature sensor based on MZ interference. The temperature sensor adopted a new MZ interference structure, which overcome the shortcomings of the small size accuracy of the flat optical waveguide. At the same time, the sensor used a frequency stabilized laser that was based on a Bragg grating fiber. The experimental results showed that the optical waveguide temperature sensor had a resolution of 0.002 °C and measuring range of 30 °C (Supplementary Information S1).

### Supplementary Information

Supplementary Information.

## Data Availability

Data underlying the results presented in this paper are not publicly available at this time but may be obtained from the authors upon reasonable request.
