# Supplementary material for: A new Mach–Zehnder interference temperature measuring sensor based on silica-based chip
Source: Sci Rep. 2024 Apr 15;14:8657. doi: 10.1038/s41598-024-59447-z (PMC11018610; doi:10.1038/s41598-024-59447-z)
Supplement: Supplementary file 1 — Supplementary Information. [file 41598_2024_59447_MOESM1_ESM.docx]

**Supplementary**

**A new Mach-Zehnder interference temperature measuring sensor based on Silica-based chip**

Guoqiang Li^1^, Tao Li^2^, Yongfang Liu^3,*^, Yuanjin Zheng^1,*^

^1^School of Electrical and Electronic Engineering, Nanyang Technological University, Nanyang Avenue, 639798, Singapore

^2^Institute of Novel Semiconductors, Institute of Crystal Materials, State Key Laboratory of Crystal Materials, Shandong University, Jinan 250100, China

^3^Shanghai Advanced Research Institute, Chinese Academy of Sciences, Shanghai 201412, China

*E-mail: yjzheng@ntu.edu.sg, liuyongfang@sari.ac.cn

### **Manufacturing of Mach-Zehnder interference**

### The Mach-Zehnder interference optical waveguide chip is processed by Shijia Photon Technology Co., Ltd. The solution uses SiO_2_ substrate to form a waveguide layer on it. Figure 3 shows the manufacturing process of optical waveguides.


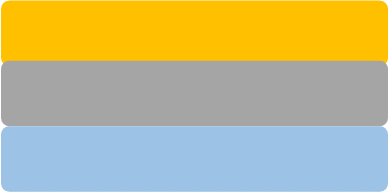

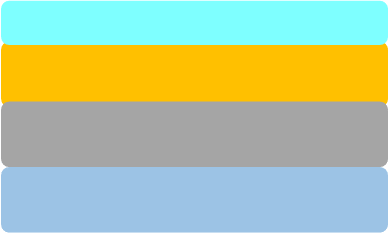

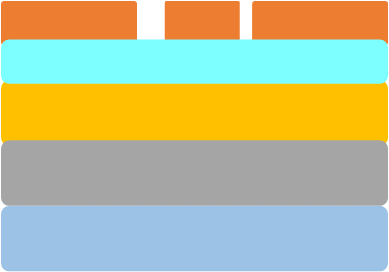

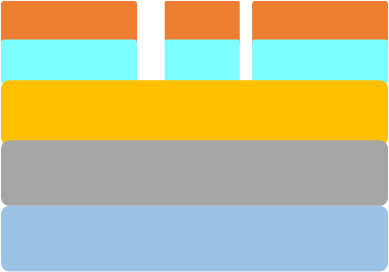

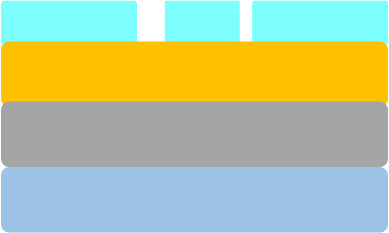

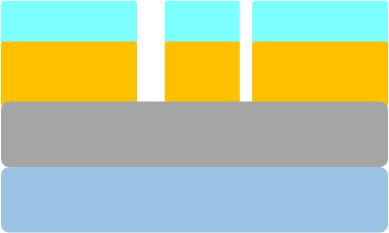

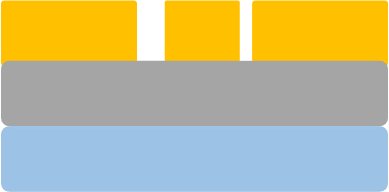

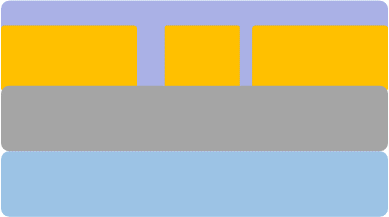


(a)

(c)

(b)

(d)

(g)

(e)

(f)

(h)


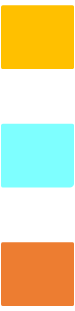

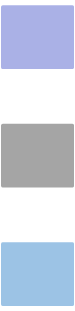


Core

Bottom Clad

Substrate

Mask

PR

Above Clad

### **Figure 3.** The manufacturing process of Mach Zehnder interference chip.

### In Figure 3, the formation of Core & Bottom Clad in step (a) is a key step in the manufacturing of optical waveguide chips, mainly including material selection, coating, curing, and subsequent processing. Firstly, it is necessary to select suitable optical materials and bottom coating materials. The selection of these materials is based on factors such as their optical properties, thermal stability, mechanical strength, and processing performance. For the Core layer, it is usually necessary to have a high refractive index to effectively guide light waves; The Bottom Clad layer needs to have a lower refractive index to ensure the propagation of light waves in the Core layer. Here, SiO2 based waveguides are used, with substrate refractive index of 1.4444 and cladding of 1.4421. Next, these two materials are respectively coated on the substrate through coating technology. During this process, it is necessary to control the uniformity and thickness of the coating to ensure the performance of the optical waveguide. After coating, it is necessary to perform thermal curing treatment on the material to form a stable structure on the substrate. Finally, perform subsequent processing on the formed Core&Bottom Clad, including cleaning, inspection, and leveling. These processing steps aim to eliminate possible defects and contamination during the processing, and improve the quality and reliability of optical waveguides.

### In step (b), make a mask. Mask is a printing like process that plays a role in protecting sensitive areas in chip manufacturing. In step (c), apply photoresist (PR) and ensure uniform coverage of the photoresist through spin coating technique. Ultraviolet light is irradiated onto the photoresist through the mask of the lithography machine, forming an exposure area corresponding to the design pattern. After exposure, the silicon wafer undergoes a development process. The developer will dissolve the photoresist portion that has been exposed to ultraviolet light, while the unexposed portion is retained. In this way, the graphics of the chip design will be displayed on the photoresist. In step (d), perform the etching process. The etching solution will dissolve the unprotected portion of the silicon wafer by the photoresist, forming a depression on the Mask that corresponds to the design pattern. In step (e), clean and inspect to remove photoresist residue and impurities generated by etching. In step (f), the Hard mask is used as the protective layer to etch the Core layer, accurately removing unnecessary Core materials, and forming the desired optical waveguide structure. The advantage of using Hard mask to etch the Core layer is that it can provide higher etching accuracy and stability, especially suitable for the manufacturing of complex and fine optical waveguide structures. In step (g), peel off the hard mask. Before starting the stripping process, ensure that the working environment is clean and dust-free to avoid introducing additional pollution or damage. In step (h), Above clad is formed. Covering and protecting the already formed waveguide structure, ensuring stable transmission of light waves within the waveguide, while preventing the influence of external environment on the performance of the waveguide. The upper cladding material here is SiO2. To ensure the transmission efficiency of light waves within the upper cladding, it is necessary to smooth the surface of the upper cladding. After completing the formation of the upper layer, thoroughly clean the chip to remove any residues and pollutants that may occur during the deposition process. Finally, the manufactured chips are tested.
